# Supplementary material for: Coupling the Cardiac Voltage-Gated Sodium Channel to Channelrhodopsin-2 Generates Novel Optical Switches for Action Potential Studies
Source: Membranes (Basel). 2022 Sep 20;12(10):907. doi: 10.3390/membranes12100907 (PMC9607247; doi:10.3390/membranes12100907)
Supplement: Supplementary file 1 [file membranes-12-00907-s001.zip › membranes-1859283-supplementary.pdf]

Construction of Na_v1.5/ChR2 heteromers

To construct Na_v1.5-CC-1, we first removed the YFP stop codon in pEYFP-hH1 [1] by mutagenesis PCR. The corresponding primer was designed to incorporate triplet TTA instead of TAA as well as palindromic sequences for the restriction sites *Pac*I and *Not*I. Both sites in the respectively modified pEYFP-hH1 plasmid were used to insert the ChR2-ChR2 dimer, amplified by PCR from pGEM-β1-ChR2-ChR2 [2]. Forward and reverse primers, containing *Pac*I and *Not*I sequences, respectively, were designed to allow also for amplification of the flanking linker GGGS-GGGS-GGGS ($[GGGS]_3$), and the transmembrane region A_2 to N_{103} of the β subunit of the human H^+/K^+-ATPase ($hβHK_{A2-N103}$; accession number NM_000705). The resulting full-length Na_v1.5-CC-1 sequence was finally inserted in two separate cloning steps as *Bcl*I/*Xba*I and *Xba*I/*Hind*III fragments into the *BamH*I/*Hind*III sites of pGEMHEnew [3]. The resulting plasmid, pGEM-Na_v1.5-YCC, was expected to encode the following heteromeric channel protein: $Na_v1.5_{M1-V2016}$-$YFP_{M1-K239}$-[LINTGVQVAE]-$[GGGS]_3$-$hβHK_{A2-N103}$-$[GGGS]_3$-$ChR2/T159C_{N24-V309}$-GGGSR$[GGGS]_2$-$hβHK_{A2-N103}$-$[GGGS]_3$-$ChR2/T159C_{N24-V309}$ (see Na_v1.5-CC-1 in Fig. 1).
For the N-terminal linkage of the ChR2 dimer to human Na_v1.5, leading to CC-Na_v1.5, we first coupled EGFP in-frame to the Na_v1.5 start codon by recombinant PCR. The full-length EGFP-Na_v1.5 fragment was inserted into the *Hind*III/*Not*I sites of pcDNA3, resulting in plasmid pcDNA-GFP-hNa_v1.5. As the next step, the ChR2 sequence was amplified by PCR from plasmid pGEM-β1-ChR2-YFP [2]. The corresponding forward primer annealed in the upstream $hβHK_{A2-N103}$ sequence and contained the *Hind*III and *Pac*I sites for the subsequent insertion of the second ChR2 sequence. The reverse primer annealed in the 3'-sequence of the Golgi-to-plasma membrane trafficking signal (peptide [KSRITSEGEYIPLDQIDINVVDTSSR]) [4] and the adjacent first YFP triplets. The resulting 1.4 kb PCR fragment was fused in-frame by a recombinant PCR step to the start codon of EGFP in pcDNA-GFP-hNa_v1.5, leading to pcDNA-GFP-hNa_v1.5/PacC. In a third cloning step, the ChR2 sequence was first amplified by a single PCR step from pGEMHE-ChR2-T159C::YFP [5,6]. Forward and reverse primers contained *Hind*III and *Pac*I sites, respectively, so that the amplified ChR2 sequence could be coupled in-frame to the ChR2-GFP-hNa_v1.5 sequence of pcDNA-GFP-hNa_v1.5/PacC. The resulting plasmid pcDNA-ChR2-ChR2-GFP-hNa_v1.5 contained the full-length CC-Na_v1.5 cDNA sequence. For cRNA production, we had to insert this 11.4 kb fragment into the *Hind*III/*Not*I sites of pGEMHEnew. The CC-Na_v1.5 amino sequence in the resulting plasmid pGEM-ChR2-ChR2-GFP-hNa_v1.5 is as follows: $ChR2/T159C_{M1-V309}$-[LINGGS]-$hβHK_{A2-N103}$-$[GGGS]_3$-$ChR2/T159C_{D2-V309}$-[KSRITSEGEYIPLDQIDINVVDTSSR]-$EGFP_{M1-K239}$-$Na_v1.5_{M1-V2016}$ (see CC-Na_v1.5 in Fig. 1).

To obtain Na$_v$1.5-CC-5, we first removed the ChR2 stop codon in pTSV40G-hNa$_v$1.5-ChR2, encoding fusion channel hNa$_v$1.5-ChR2$_{D2\text{-}V309}$, by mutagenesis PCR (for pTSV40G-hNa$_v$1.5-ChR2, encoding the similar variant hNa$_v$1.5-ChR2$_{N24\text{-}V309}$, see [2]). The corresponding forward primer annealed in the hNa$_v$1.5 sequence upstream to the unique *Tth111*I site. The reverse primer was designed to incorporate triplet TTA instead of TAA as well as sequences for the restriction sites *Pac*I and *Not*I. The 1.7 bp PCR fragment was inserted into the *Tth111*I/*Not*I sites of pTSV40G-hNa$_v$1.5-ChR2, resulting in pTSV40G-hNa$_v$1.5-ChR2/Pac. Next, we amplified by PCR the coding region for VQVAE-[GGGS]$_3$-hβHK$_{A2\text{-}N103}$-[GGGS]$_3$-ChR2/T159C$_{N24\text{-}V309}$-[KSRITSEGEYIPLDQIDINVVDTSSR]-YFP$_{M1\text{-}K239}$-[SRFCYENEV] from pGEM-β1-ChR2-YFP [2]. The Golgi-to-plasma membrane trafficking signal peptide [KSRITSEGEYIPLDQIDINVVDTSSR] [4] and the ER export motif [SRFCYENEV] [7] were previously incorporated into the β1-ChR2-YFP construct in order to increase the channel number in the plasma membrane [2]. The corresponding forward primer contained a *Pac*I site; the reverse primer annealed in the 3'-non-coding region of the β-globin sequence of pGEMHEnew and contained a *Not*I site. The resulting 2.3 kb PCR fragment was digested with *Pac*I and *Not*I, and inserted into the same sites of pTSV40G-hNa$_v$1.5-ChR2/Pac. The resulting plasmid, pTSV40G-hNa$_v$1.5-ChR2-ChR2-YFP, was used to produce the Na$_v$1.5-CC-5 cRNA, and finally the following channel protein: Na$_v$1.5$_{M1\text{-}V2016}$-[GGGS]$_3$-hβHK$_{A2\text{-}N103}$-[GGGS]$_3$-ChR2/T159C$_{D2\text{-}V309}$-[LINVQVAE]-[GGGS]$_3$- hβHK$_{A2\text{-}N103}$-[GGGS]$_3$-ChR2/T159C$_{N24\text{-}V309}$-[KSRITSEGEYIPLDQIDINVVDTSSR]-YFP$_{M1\text{-}K239}$-[SRFCYENEV] (see Na$_v$1.5-CC-5 in Fig. 1).

For the construction of Na$_v$1.5-CC-4, the ER export motif [SRFCYENEV] of Na$_v$1.5-CC-5 was deleted in pTSV40G-hNa$_v$1.5-ChR2-ChR2-YFP by recombinant PCR. The amino sequence, encoded by the resulting plasmid pTSV40G-hNa$_v$1.5-ChR2-ChR2-YFP0, is as follows: Na$_v$1.5$_{M1\text{-}V2016}$-[GGGS]$_3$-hβHK$_{A2\text{-}N103}$-[GGGS]$_3$-ChR2/T159C$_{D2\text{-}V309}$-[LINVQVAE]-[GGGS]$_3$-hβHK$_{A2\text{-}N103}$-[GGGS]$_3$-ChR2/T159C$_{N24\text{-}V309}$-[KSRITSEGEYIPLDQIDINVVDTSSR]-YFP$_{M1\text{-}K239}$ (see Na$_v$1.5-CC-4 in Fig. 1).

To obtain Na$_v$1.5-CC-3, we used plasmid pTSV40G-hNa$_v$1.5-ChR2-ChR2-YFP as a PCR template to amplify a 1.4 kb fragment, encoding the following protein sequence: TGVQVAE-[GGGS]$_3$- hβHK$_{A2\text{-}N103}$-[GGGS]$_3$-ChR2/T159C$_{N24\text{-}V309}$-[KSRITSEGEYIPLDQIDINVVDTSSR]-MVSKG. Forward and reverse primers contained restriction sites *Pac*I and *Xba*I, respectively, so that the resulting PCR product could be inserted in-frame into the same sites of pGEM-Na$_v$1.5-YCC (see cloning for Na$_v$1.5-CC-1 above). The resulting plasmid, pGEM-Na$_v$1.5-YC-ts-C, allowed for the production of the Na$_v$1.5-CC-3 cRNA, encoding the following fusion channel: Na$_v$1.5$_{M1\text{-}V2016}$-YFP$_{M1\text{-}K239}$-[LINTGVQVAE]-[GGGS]$_3$-hβHK$_{A2\text{-}N103}$-[GGGS]$_3$-ChR2/T159C$_{N24\text{-}V309}$-[KSRITSEGEYIPLDQIDINVVDTSSR]-[GGGS]$_2$-hβHK$_{A2\text{-}N103}$-[GGGS]$_3$-ChR2/T159C$_{N24\text{-}V309}$ (see Na$_v$1.5-CC-3 in Fig. 1).

To obtain Na_v1.5-CC-2, we inserted by a recombinant PCR approach the sequence for the Golgi-to-plasma membrane trafficking peptide (see above) in-frame between the Na_v1.5 and YFP sequences in plasmid pGEM-Na_v1.5-YCC (see cloning for Na_v1.5-CC-1). The Na_v1.5-CC-2 amino sequence in the resulting plasmid pGEM-hNa_v1.5-ts-YCC is as follows: $Na_v1.5_{M1\text{-}V2016}$-[KSRITSEGEYIPLDQIDINVVDTSSR]-$YFP_{M1\text{-}K239}$-[LINTGVQVAE]-$[GGGS]_3$-$h\beta HK_{A2\text{-}N103}$-$[GGGS]_3$-$ChR2/T159C_{N24\text{-}V309}$-GGGSR$[GGGS]_2$-$h\beta HK_{A2\text{-}N103}$-$[GGGS]_3$-$ChR2/T159C_{N24\text{-}V309}$ (see Na_v1.5-CC-1 in Fig. 1).

References

1. Zimmer, T.; Biskup, C.; Dugarmaa, S.; Vogel, F.; Steinbis, M.; Bohle, T.; Wu, Y.S.; Dumaine, R.; Benndorf, K. Functional expression of GFP-linked human heart sodium channel (hH1) and subcellular localization of the a subunit in HEK293 cells and dog cardiac myocytes. *J Membr Biol* **2002**, *186*, 1-12.
2. Walther, F.; Feind, D.; Vom Dahl, C.; Muller, C.E.; Kukaj, T.; Sattler, C.; Nagel, G.; Gao, S.; Zimmer, T. Action potentials in Xenopus oocytes triggered by blue light. *The Journal of general physiology* **2020**, *152*, doi:10.1085/jgp.201912489.
3. Liman, E.R.; Tytgat, J.; Hess, P. Subunit stoichiometry of a mammalian K+ channel determined by construction of multimeric cDNAs. *Neuron* **1992**, *9*, 861-871.
4. Hofherr, A.; Fakler, B.; Klocker, N. Selective Golgi export of Kir2.1 controls the stoichiometry of functional Kir2.x channel heteromers. *Journal of cell science* **2005**, *118*, 1935-1943, doi:10.1242/jcs.02322.
5. Berndt, A.; Schoenenberger, P.; Mattis, J.; Tye, K.M.; Deisseroth, K.; Hegemann, P.; Oertner, T.G. High-efficiency channelrhodopsins for fast neuronal stimulation at low light levels. *Proc Natl Acad Sci U S A* **2011**, *108*, 7595-7600, doi:10.1073/pnas.1017210108.
6. Ullrich, S.; Gueta, R.; Nagel, G. Degradation of channelopsin-2 in the absence of retinal and degradation resistance in certain mutants. *Biological chemistry* **2013**, *394*, 271-280, doi:10.1515/hsz-2012-0256.
7. Stockklausner, C.; Ludwig, J.; Ruppersberg, J.P.; Klocker, N. A sequence motif responsible for ER export and surface expression of Kir2.0 inward rectifier K(+) channels. *FEBS Lett* **2001**, *493*, 129-133.

Table S1. Supplementary statistical AP and oocyte parameters. Oocytes with sub-threshold depolarization were excluded.

Channel constructs	Peak Na^+ current (μA)	Peak photocurrent (μA)	K^+ current at 10 mV (μA)	K^+ current at 30 mV (μA)	Oocyte features: Resting potential (mV)	Oocyte features: Membrane resistance (MΩ)	Number of oocyte batches	Number of measurements
Na_v1.5-CC-2	15.58±0.66	0.37±0.03	-	-	-20.58±1.02	0.96±0.07	8	12
Na_v1.5-CC-5	18.02±0.53*	0.22±0.02*	-	-	-25.67±0.40	1.03±0.09	4	12
Na_v1.5-CC-2 + K_v1.2	15.99±0.95	0.62±0.04*	3.00±0.25	4.25±0.39	-30.80±0.53*	0.79±0.04*	5	15
Na_v1.5-CC-2 + hERG	13.49±0.47*	0.47±0.02*	1.97±0.13	2.04±0.13	-49.94±0.62*	0.52±0.02*	5	16
Na_v1.5-CC-ΔKPQ	11.27±0.53*	0.51±0.03*	-	-	-16.17±0.74*	0.94±0.05	6	18

* indicates $p<0.05$ *versus* Na_v1.5-CC-2

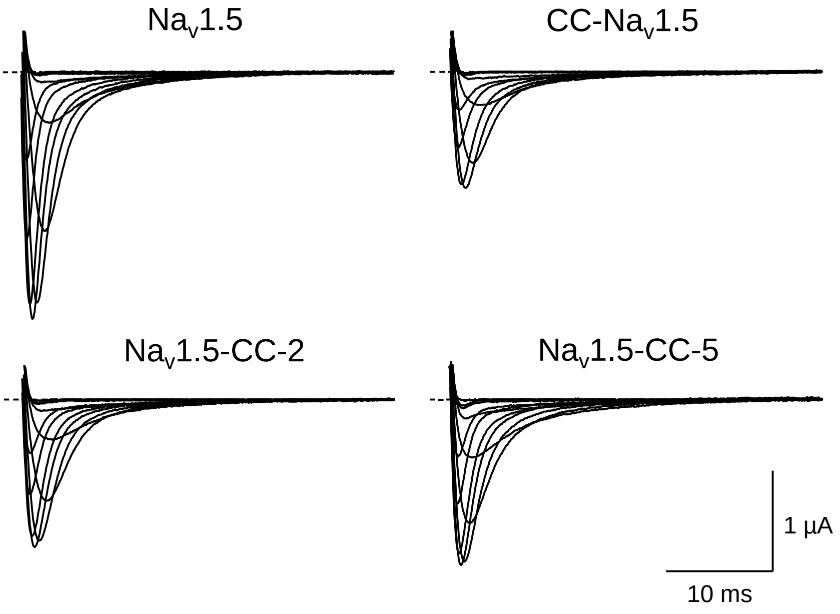

Figure S1. Representative Na^+ current families of wild-type Na$_v$1.5 and three fusion channels. Whole-cell currents were recorded three days after cRNA injection at a test potential between -80 and -45 mV (holding potential -100 mV). For the injected cRNA amount and detailed statistics on Na^+ channel kinetics see Table 2.
